# PROTOCOL: Psychological and psychosocial determinants of COVID Health Related Behaviours (COHeRe): A suite of systematic reviews and an evidence and gap map

**DOI:** 10.1002/cl2.1219

**Published:** 2022-02-03

**Authors:** Jennifer Hanratty, Ciara Keenan, Sean O'Connor, Sarah Miller, Declan Bradley, Martin Dempster

**Affiliations:** ^1^ School of Psychology Queen's University Belfast UK; ^2^ School of Social Sciences, Education and Social Work Queen's University Belfast UK; ^3^ School of Medicine, Dentistry and Biomedical Sciences Queens University Belfast UK

## Abstract

This is the protocol for a Campbell systematic review. The objectives are as follows: we intend to identify and synthesise the existing evidence (published and unpublished) on malleable psychological and psychosocial factors that determine uptake and adherence to behaviours that can reduce the risk of infection or transmission of COVID‐19.

## 1 BACKGROUND

### 1.1 Introduction

#### 1.1.1 The problem, condition or issue

The second severe acute respiratory coronavirus (SARS‐CoV‐2) emerged in late 2019 and spread rapidly around the globe (Cucinotta & Vanelli, 2020; Wu et al., 2020). The pandemic of COVID‐19 disease, caused by SARS‐CoV‐2, has resulted in illness, deaths and societal disruption. Around the world, societies have implemented control measures to reduce transmission of the virus. Individual behaviour change is crucial to the success of these measures though reducing the frequency of social contacts, mitigating the risk of those social contacts and reducing the amount of time that infectious people are at large. Vaccine rollout began in high income countries as of December 2020 but until vaccines are delivered at scale globally, behavioural measures to reduce the spread remain vital (Girum et al., 2021; Michie & West, 2020).

The behaviours to reduce the risk of catching or spreading SARS‐CoV‐2 that are globally recommended are: hand washing or use of hand sanitizer, wearing masks/face coverings, physical distancing, social distancing, isolation/quarantine, respiratory hygiene, cleaning surfaces, avoiding touching the t‐zone as well as other composite measures that include the above. The evidence for the effectiveness of these measures has been established during previous pandemics of similar serious viral respiratory infections such as H1N1, SARS and MERS (Flumignan et al., 2020; Jefferson et al., 2020; Seto et al., 2003; Warren‐Gash et al., 2013; Webster et al., 2020; West et al., 2020). These behaviours are also referred to as non‐pharmaceutical interventions (NPIs), physical measures or behavioural protections.

#### 1.1.2 Why it is important to develop the systematic reviews and EGM

We will identify and map all existing research evidence that primarily focuses on health protective behaviours in the context of SARS‐CoV‐2. In any future severe viral outbreaks health protective behaviours remain vital to reduce risk of infection and transmission. Health protective behaviours cannot be effective if they are not adopted widely and consistently. Despite evidence on the effectiveness of control measures, there is still variation in their uptake at the level of national and local government policy and at an individual level. Variables such as a person's health beliefs, social support, culture, social norms, etc. can all influence the likelihood of someone undertaking a health behaviour. To develop appropriate public health interventions to improve uptake and adherence to these behaviours, including effective messaging, we need to understand the malleable factors that influence behaviour.

The effectiveness of behaviour change interventions will be determined, to some extent, by how they address the psychological and psychosocial variables that influence behaviour. To optimise public health intervention, we need to know which specific variables are most likely to influence the target behaviours in this context. Evidence gathered in the context of COVID‐19 and generated during and following previous outbreaks of similar communicable serious respiratory infections can inform who, when and under what circumstances people do or do not adopt recommended preventive behaviours. The proposed project seeks to synthesise the determinants of variability in the adoption and maintenance of protective behaviours at an individual level.

#### 1.1.3 Existing EGMs and/or relevant systematic reviews

There are a number of related published and ongoing reviews on individual determinants of health‐related behaviours but none with the scope and rigour of our proposed project. Using robust search, retrieval, and methodological approaches to minimise potential sources of research bias with the proposed systematic reviews and evidence and gap map (EGM), we will summarise all the existing and emerging evidence in one place, for the first time. To our knowledge, no EGMs exist that assess the available evidence on health‐protective behaviours in the context of SARS‐CoV‐2.

## 2 OBJECTIVES

We intend to identify and synthesise the existing evidence (published and unpublished) on malleable psychological and psychosocial factors that determine uptake and adherence to behaviours that can reduce the risk of infection or transmission of COVID‐19. The specific behaviours of interest are as follows:

- Handwashing
- Wearing masks/face coverings
- Physical Distancing
- Social Distancing
- Isolation/quarantine
- Respiratory hygiene
- Cleaning surfaces
- Avoiding t‐zone
- Other composite measures that include the above.

In addition to this, we will create a live, searchable and publicly available EGM containing both primary and secondary research studies, that includes a wider body of evidence on any determinants, not limited to only malleable psychological and psychosocial factors. The EGM is an important addition to this project, as this will likely be the point of engagement for many key stakeholders. EGMs allow users to see, at a glance, where research exists and where there are gaps. This is important as funders can see where there is a saturation of evidence and redirect much‐needed resources towards important gaps. Researchers can see where there is potential to research and identify the risk of duplicating effort. Public health bodies can see where there is evidence and the quality of this evidence. Members of the public can make informed decisions about their health choices.

## 3 METHODS

### 3.1 Eligibility criteria

#### 3.1.1 Types of studies

The systematic reviews will contain studies that quantify the relationship between a potential determinant and one or more of the behaviours of interest. Study designs will include observational studies (both retrospective and prospective) and experimental studies that measure and report malleable psychological and psychosocial determinants and behaviours at an individual level. We will not include narrative reviews, modelling studies, letters, editorials, opinion pieces, news, commentaries, or any other publications that do not report primary data. The EGM will have broader eligibility criteria than the systematic reviews, in that it will also identify and include systematic reviews and qualitative studies and studies that measure nonmalleable determinants, such as demographics.

#### 3.1.2 Population

Across both the systematic reviews and EGM, the population of interest is members of the general public, of any age.

Within the group of studies of the general public, we will include studies on specific groups of people that may be at increased risk of catching the virus, for example, people who work in essential retail services. Similarly, will we include studies of specific patient groups at increased risk of becoming seriously ill if infected, for example, those with existing chronic respiratory disorders.

However, we will not include studies on health care workers (HCWs), defined as someone who works in a hospital or health care setting or delivers health care in the community. This population typically have, or should have additional knowledge, training and resources to support the adoption of behaviours to mitigate against the increased risk of exposure to infectious diseases. A rapid review on barriers and facilitators to HCWs adherence to infection prevention and control guidelines has been published Houghton et al. (2020). Should studies include both HCWs and the public, we will only include these studies if data on the public is presented separately from data on healthcare workers.

#### 3.1.3 Context

Across both the systematic reviews and EGM, we will include only those studies which were conducted during the ongoing COVID‐19 pandemic. We will include studies from Jan 2020 until the date of the final search.

#### 3.1.4 Exposure/determinants

The exposure in this review refers to any potential determinant of one or more of the behaviours of interest described above. Within the EGM this can include both malleable and nonmalleable determinants. The systematic reviews will be focused on the subset of studies that measure psychological or psychosocial determinants. To be included in the systematic reviews, determinants must be malleable factors that could, theoretically, be changed by a public health intervention. We anticipate that determinants examined in the literature may include, but not be limited to:

- Cognitive factors such as knowledge of the transmission, knowledge of protective behaviours, perceived severity of the disease, perceived susceptibility.
- Affective factors such as worry or fear of catching or transmitting the virus.
- Psychosocial factors, such as perceived social support, perceived health status.
- We also consider other factors that may be influenced by public policy such as access to paid sick leave, access to childcare, financial support for self‐isolation.

We deliberately do not approach this synthesis with a particular framework or theory of health behaviour driving the selection of potential determinants or synthesis decisions. Instead, we seek to produce a list of potential malleable determinants that could be changed by public health intervention. That way, we can provide a summary of the published evidence on which factors do and do not determine behaviours of interest. For those factors that relate to behaviours of interest, we will quantify the strength of the relationship through meta‐analysis.

#### 3.1.5 Comparator: Absence of determinant, or lower degree of the determinant

Comparators will be the absence of the determinant (compared to its presence) or, where a determinant is presented as a continuous measure, then analysis will be based on correlation between behaviours and determinants. See section Measures of treatment effect for further details on the treatment of different types of data.

#### 3.1.6 Measurement of determinants

We will include studies that measure determinants at an individual level only.

We will not include studies where a determinant is measured at a group level, for example, country level data on number of infected cases.

We will include studies on self‐reported or observed determinants. Self‐reports may include actual or perceived determinants, for example ‘risk of contracting the virus' could be measured by quantifying actual risk based on individual circumstances and behaviour or through self‐reported perceived risk. We will include determinants measured through self/other report and/or observation, so long as this measurement is at an individual level.

We will carefully consider issues of measurement and the potential differences in effect estimates of actual, intended, perceived or hypothetical determinants when making decisions on what effect sizes, from which studies, can be reasonably combined in meta‐analysis.

#### 3.1.7 Outcomes: Behaviours of interest

This review seeks to synthesise evidence on determinants of the commonly recommended behaviours to mitigate human‐to‐human spread of COVID‐19 as described by West et al. (2020). Table 1 describes in detail the behaviours of interest in this review.

**Table 1:** Behaviours to mitigate the spread of COVID‐19

Behaviours	Description of behaviours
Handwashing	Washing hands more frequently with soap and water or the use of hand sanitizer if handwashing facilities are not available.
Masks/face covering	Wearing any type of mask or face covering. This can include medical grade masks, face shields, homemade masks or covering face with a scarf.
Physical Distancing	Maintaining the recommended distance from others when physically present. The recommended distance varies by setting but is typically in the region of 1 to 3 m.
Social Distancing	Minimising social contact with those outside of your own household. This is a very broad category and includes working from home, avoiding crowded places, only leaving home when necessary (e.g., to purchase food or medicines) and not socialising with others in your own home or garden.
Isolation/quarantine	Self‐isolation and/or quarantine refers to keeping separate from all other people either because you have or are suspected to have the virus. Self‐isolation is typically voluntary but often recommended by the government/health authorities. Quarantine is typically enforced in either a mandated setting, one's own home, or temporary accommodation for those in travelling away from home.
Respiratory hygiene	Includes tissue hygiene, which means using a tissue to cover nose and mouth when coughing, sneezing or blowing your nose and immediately disposing of the tissue. When tissues are not available coughing/sneezing into your elbow and not your hands.
Cleaning surfaces	Disinfecting high touch surfaces in home and office/retail/public spaces or items brought into the home.
Avoiding t‐zone	Avoiding touching your face specifically the t‐zone; eyes, nose and mouth.
Other	Other analogous relevant behaviours or aggregate measures of multiple relevant behaviours.

Other behaviours may be recommended in different countries/regions and so the behaviours of interest in this review will not be an exhaustive list of behaviours that might mitigate the spread of COVID‐19. They are, however, the most commonly recommended behaviours globally.

#### 3.1.8 Measurement of behaviours

We will include studies on actual behaviour, intended behaviour or hypothetical behaviour. We will include behaviours measured through self/other report and/or observation of actual behaviour, so long as this measurement is at an individual level.

As with determinants, we will carefully consider issues of measurement and the potential differences in effect estimates of actual, intended or hypothetical behaviour when making decisions on what effect sizes, from which studies, can be reasonably combined in meta‐analysis.

### 3.2 EGM: Definition and purpose

As indicated, an EGM will also be utilised. EGMs are a tool to prioritise research needs and to support evidence‐informed practice and policy decisions.

This part of the project will follow five steps:

(1) Scoping and development of the EGM framework
(2) systematic and comprehensive searches
(3) screening for eligibility (i.e., title &abstract, then full text)
(4) data extraction
(5) high‐level quality appraisal
(6) and analysis (according to the predefined inclusion/exclusion criteria).

### 3.3 Framework development and scope

We will follow the standard EGM framework as a matrix, with rows containing the behaviour (i.e., Handwashing, wearing masks/face coverings, physical Distancing, etc.) and columns containing information regarding the determinants (i.e., Cognitive factors, affective factors, etc.). Filters will also be added to the EGM. The framework and filters used will be developed using our existing knowledge of the extant evidence and through engaging with consumers and expert advisors. The engagement of consumers/stakeholders will be supported by the Cochrane Consumer group. The consumers will be people who are already engaged with the Cochrane COVID Consumer Group.

### 3.4 Search methods and sources

#### 3.4.1 Search strategy

We have developed and piloted a search strategy with the guidance of a Campbell information retrieval specialist (C. K.). Systematic reviews and EGMs are underpinned by a systematic search of the literature, using various literature sources including electronic databases, web searches, conference proceedings, government reports and other repositories of literature. To ensure that the literature contained in the reviews and map is relevant and useful to key stakeholders, it is important that the literature retrieval methods follow high‐quality standards, therefore, all searches will be conducted and reported following Campbell Collaboration guidelines (White et al., 2020).

The search strategy has been built around three concepts of interest including (1) context (terms relating to COVID‐19), (2) behaviours of interest and (3) terms related to psychological and psychosocial determinants of COVID Health‐Related Behaviours and adherence or compliance with recommended behaviours, to capture both malleable and nonmalleable determinants. For concept one, an innovative and tested COVID‐19 search strategy has been developed for use by NICE information specialists and has been updated as recently as 21 June 2021 (Levay & Finnegan, 2021). An example of the search string has been piloted in Medline (Ovid) and is presented in Table 2.

**Table 2:** Medline (Ovid) search strategy

Ovid MEDLINE(R) ALL < 1946 to September 03, 2021>		
1	SARS‐CoV‐2/or COVID‐19/	103,591
2	(corona* adj1 (virus* or viral*)).ti,ab.	2364
3	(CoV not (Coefficien* or “co‐efficien*” or covalent* or Covington* or covariant* or covarianc* or “cut‐off value*” or “cutoff value*” or “cut‐off volume*” or “cutoff volume*” or “combined optimi?ation value*” or “central vessel trunk*” or CoVR or CoVS)).ti,ab.	51,911
4	(coronavirus* or 2019nCoV* or 19nCoV* or “2019 novel*" or Ncov* or “n‐cov” or “SARS‐CoV‐2*" or “SARSCoV‐2*” or SARSCoV2* or “SARS‐CoV2*” or “severe acute respiratory syndrome*” or COVID*2).ti,ab.	181,470
5	or/1‐4	187,096
6	limit 5 to yr = "2020‐Current"	173,962
7	(6 and english.lg.) not (letter or historical article or comment or editorial or news).pt. not (Animals/not humans/)	134,173
8	(Mask or masks or face?mask* or Face cover*).ti,ab.	42,975
9	(face adj2 (shield or shields)).ti,ab.	414
10	(((Hand or hands) adj2 hygiene) or Handwash* or (Wash* adj2 hand*)).ti,ab.	11,132
11	(hand adj1 clean*).ti,ab.	256
12	(hand adj2 saniti*).ti,ab.	683
13	(hand adj2 disinfect*).ti,ab.	783
14	Respiratory hygiene.ti,ab.	79
15	Respiratory etiquette.ti,ab.	27
16	((cough* or sneeze*) and (sleeve or arm or elbow or tissue or etiquette)).ti,ab.	2752
17	(tissue and (dispose or disposal or bin or hygiene)).ti,ab.	3414
18	universal hygiene.ti,ab.	10
19	Social Isolation/or Patient Isolation/	19,284
20	(self‐isolate or self‐isolation or self‐isolating).ti,ab.	724
21	(mass adj2 (behav* or gather*)).ti,ab.	1690
22	(social distance or social distancing).ti,ab.	6625
23	stay at home.ti,ab.	1465
24	stay home.ti,ab.	314
25	((work* adj2 home) or telecommute or telework* or (remote* adj2 work*)).ti,ab.	5262
26	(Physical adj2 distanc*).ti,ab.	2595
27	(touch* and (mouth or mouths or face or faces or nose or noses or t‐zone)).ti,ab.	1635
28	disinfect*.ti,ab.	31,760
29	lockdown.ti,ab.	8167
30	quarantine.ti,ab.	7821
31	(nonpharmaceutical or non‐pharmaceutical).ti,ab.	1831
32	(school closure or close school* or school closing).ti,ab.	389
33	or/8‐32	140,404
34	limit 33 to yr = “2020‐Current”	34,955
35	(34 and english.lg.) not (letter or historical article or comment or editorial or news).pt. not (Animals/not humans/)	31,455
36	7 and 35	20,298
37	exp Knowledge/	12,323
38	exp Health knowledge, Attitudes, Practice/	119,567
39	(Knowledg* or Personal* or Attitude* or Practice* or Habit* or belie* or Behav* or Need* or prevent* or Compliance or comply* or complied or Perception* or Protect* or Predict* or view* or barrier* or facilitator* or readiness or prepar* or ability* or insight or proficien* or procedur* or adher*).ti,ab.	10,617,318
40	or/37‐39	10,635,825
41	7 and 35 and 40	14,859

##### Electronic databases

Based on the Queens's University Belfast database subscriptions, we will search the following key information sources to locate relevant primary research:

- Medline ALL (Ovid)
- Child Development & Adolescent Studies (EBSCOhost)
- ERIC (EBSCOhost)
- PsycInfo 1806‐present (OVID)
- CINAHL Plus (EBSCOhost)
- Web of Science Core Collection (the QUB subscription includes SCI‐expanded, SSCI, A&HCI, CPCI‐S, CPCI‐SSH, ESHI).

To locate relevant secondary research for inclusion in the EGM, we will search the following information resources:

- The Social Care Institute for Excellence (SCIE)
- The Cochrane Library
- Epistemonikos Covid‐19 evidence platform
- Norwegian Institute of Public Health living maps
- EPPI – centre
- COVID‐END.

##### Other sources

We will search for Grey literature across multiple sources. Grey literature is that which is not published, not peer reviewed, and not easily accessible. Sources of grey literature are varied and include government reports, privately and publicly funded research, conference proceedings, working papers, and posters. Some grey literature sources are captured in the Web of Science search, these include:

- Conference Proceedings Citation Index‐ Science (CPCI‐S)—1990‐present
- Conference Proceedings Citation Index‐ Social Science & Humanities (CPCI‐SSH)—1990‐present.

We will attempt to locate additional grey literature by searching sources such as the following:

- Google Scholar (We will search https://scholar.google.com/using an incognito browser and the following strategy: (coronavirus | “2019 nCoV”| “2019 novel”| “2019 nCoV”| “2019 nCoV”| CoV | “COVID 19” |COVID19 | “COVID 19”| ncov | “SARS CoV2”| “SARS CoV 2”|“severe acute respiratory syndrome Coronavirus 2”) (Psychological | Psychosocial)(behavior|behaviour) we will limit returns by ‘Since 2020’ filter and sort remaining records by relevance. We will then download the first 1000 articles (which is the upper limit set by google) using Harzing's Publish or Perish software.
- clinicaltrials.gov
- ISRCTN Registry (https://www.isrctn.com/)
- WHO International Clinical Trials Registry Platform (ICTRP) (https://www.who.int/clinical-trials-registry-platform/the-ictrp-search-portal).

And by contacting and reviewing the information of the following key organisations in the UK with proven experience on the topics related to this project:

- King's Fund (https://www.kingsfund.org.uk/)
- National Institute for Health Research (https://www.nihr.ac.uk/)
- NHS Evidence (https://www.evidence.nhs.uk/).

We considered searching ProQuest dissertations and theses, however, we realise that it unlikely that relevant doctoral theses would be complete and available in the timeframe of the virus.

We also intend to conduct a search of reference lists of previous reviews and eligible articles to identify any additional studies not identified through the electronic search. Finally, when we have complied a list of included studies we will contact key experts in the field via email (categorised as ‘key’ if they have published five or more included studies) to ask whether they are aware of any unpublished or ongoing research that might not be easily accessible to the research team.

To locate relevant grey literature for inclusion in the EGM, we will search for ongoing or unpublished reviews via:

- PROSPERO,
- Figshare and the
- Open Science Framework (OSF).

Any ongoing reviews will be checked again before completion of the project and if still unpublished 1 month before submission they will be added to a reference section titled ‘ongoing reviews’ and excluded from the map.

##### Search limits

We will not limit searches by language of publication but, due to the limited language skills of the review team, we will only include studies published in English. We will list studies in other languages that appear relevant as studies ‘awaiting classification’ and we will invite interested researchers to contact the review team if they can assist with translation and data extraction so that these studies can be included in any update of the review.

We will limit our search to exclude opinion pieces, letters, editorials and unpublished reports in databases where these limits are supported (see Table 2: lines 7 and 35). We will not use database limiters for studies on humans only as we found these limiters excluded a substantial number of potentially relevant papers not indexed as ‘human’ studies. Instead, we have opted to use an adaptation of the Cochrane search filter for human studies (line 7 and 35).

Across both the systematic reviews and EGM, we will include only those studies which were conducted during the ongoing COVID‐19 pandemic. We will include studies from Jan 2020 until the date of the final search.

### 3.5 Data collection and analysis

#### 3.5.1 Screening and study selection

Once the database searches have been conducted, results will be imported to a bibliographic reference manager where duplications of identical studies gathered from multiple sources can be removed to avoid duplication of effort.

Following this, screening will be supported by the Cochrane Crowd (Noel‐Storr et al., 2021). This platform provides a mechanism for volunteers to undertake different tasks related to the identification of studies as part of health care evidence reviews. The platform has been shown to have sufficient accuracy in terms of study identification (Gartlehner et al., 2020; Noel‐Storr, Dooley, Elliott, et al., 2021; Noel‐Storr, Dooley, Affengruber, et al., 2021; Noel‐Storr, Redmond, et al., 2021). It also provides a scalable approach, allowing it to support the screening of rapidly emerging evidence. Following successful completion of a brief training module, screeners will be asked to screen titles and abstracts against the eligibility criteria and to identity potential studies as ‘possibly relevant’ or ‘not relevant’. Each record will be screened by at least three independent screeners. Where there are any conflicts between screener decisions, these will be resolved by members of the core research team. When this initial screening of title and abstracts is complete, members of the research team will screen all potentially relevant studies at full‐text level. All screening decisions and coding of the included studies will be documented and will be made publically available via the project page on Open Science Framework (https://osf.io/hv5s3/).

#### 3.5.2 Data extraction and management

Data extraction procedures will be managed in EPPI‐Reviewer software (Thomas et al., 2010). Once eligible studies have been identified from full‐text screening, one author will extract data and complete risk of bias assessments. Any studies remaining after full‐text screening but which are subsequently identified as ineligible during data extraction will be listed as ‘excluded’. A second author will check the data extraction and risk of bias assessments on at least 20% of included papers. The two people who completed the data extraction for each study in this sample of 20% will discuss any discrepancies until they reach a consensus or, if necessary, refer to a third author to make a final decision. The full data extraction form is included in Supporting Information Appendix 1.

We will extract the following data:

- Study information: author, year, country, study design, disease, when the study was conducted, sample size.
- Population: Where recruited from, description of the population, relevant sectors, geographic location, age, sex, ethnicity, socioeconomic status, other including education level, disease status.
- Exposure and Comparator: Determinant measured, description of the measurement tool used and its quality, who measured the determinant, the type of measurement (observation, self‐report, etc.), direction of scale.
- Outcomes: Behaviour measured, description of the behaviour measurement tool used and its quality, who measured the behaviour, the type of measurement (observation, self‐report, etc.), direction of scale.
- Effects: Narrative description of the finding, effect size information or sufficient numerical data to allow us to calculate the effect size.

#### 3.5.3 Quality appraisal

We will assess methodological quality and potential for bias using the second version of the Cochrane Risk of Bias tool for any Randomised controlled trials included (Sterne et al., 2019), the ROBINS‐I tool (Sterne et al., 2016) for non‐randomised intervention studies and the JBI tools for longitudinal and cross‐sectional studies respectively (The Joanna Briggs Institute, 2017, 2020). After piloting the JBI tool on some known studies we decided to modify the tools to ensure that they are fit for our purposes (Supporting Information Appendix 2). Briefly, added items to assess whether or not the sample is representative of the population of interest in each study and changed the wording slightly, replacing condition and exposure with behaviours of interest and determinants.

We will use these tools to assign an overall ‘risk of bias’ to each study and integrate this in the narrative synthesis and accompanying tables. We will also assess the impact of removing low‐quality studies (those with high risk of bias) in sensitivity analysis and incorporate RoB into summary of findings table.

For the EGM, we will appraise the methodological quality of systematic reviews with AMSTAR‐2 (Shea et al., 2017).

##### Measures of treatment effect

As we are examining determinants of behaviour, we will extract metrics that quantify the relationship between behaviours of interest and determinants of that behaviour. We anticipate that for dichotomous data this will be odds ratios. If these summary statistics are not presented, we will seek to extract data that allows us to calculate OR (e.g., the percentage of men vs percentage women engaging in the behaviour). If rate ratios are reported these will be extracted and converted to OR for analysis. For continuous data, we will extract correlation coefficients or regression coefficients and convert these to OR for meta‐analysis. Where ordinal data is reported in included studies, for example where participants are categorised according to four age groups and behaviour compared between groups, we cannot yet say what approach will allow us to make optimal use of the available data. This is because we do not yet know if different studies will use ordinal scales/groupings that are similar enough to combine. One approach would be to select a ‘cut‐point’ and dichotomise ordinal data (Higgins et al., 2020), for example dichotomising multiple age categories into ‘age 70 and above’ and ‘under age 70’ or re‐categorising multiple income gradients into ‘above average income’ and ‘average or below average income’. Rather than selecting arbitrary cut points in advance we will endeavour to extract all data and consider the approach to meta‐analysis in light of the data available and in consideration of the most useful questions from both theoretical and policy perspectives. Where other approaches to data analysis are used, for example, analysis of covariance or structural equation modelling we will defer to Dempster, who is a chartered statistician, to decide what data can be extracted and used in meta‐analysis.

##### Unit of analysis issues

If a paper reports on multiple separate studies we will treat these as individual studies and refer to them as ‘author year S1’, ‘author year S2’, etc. If a single study is reported in multiple papers, we will only include as one study and extract information from all relevant reports.

Where there are multiple measures reported for the same outcome, we will use robust variance estimation (RVE) to adjust for effect size dependency (Hedges et al., 2010; Tipton, 2013). This technique calculates the variance between effect sizes to give the variable of interest a quantifiable standard error. It has been shown to calculate correct results with a minimum of 20–30 individual studies (Hedges et al., 2010) although it performs better with an increased quantity of studies. The correction for small samples (Tipton & Pustejovsky, 2015) will be implemented when necessary.

Where the same outcome construct is measured but across multiple time domains, such as before, during and after a pandemic, we will use RVE to allow us to include all effect sizes in analysis and conduct subgroup analysis to assess the impact of time or phase of the outbreak on behaviour. We will categorise each exposure × outcome relationship according to the time in the outbreak as follows: hypothetical, pre‐pandemic/outbreak, during pandemic/outbreak, after‐pandemic/outbreak. If studies report results over more granular periods of time then synthesis will be data‐driven (e.g., pre‐peak vs. post‐peak) and dependant on the number of studies reporting over similar time‐frames.

We have describe above the approach taken with ordinal data comparing multiple groups, such as studies reporting on behaviour/determinants relationships for low, middle and upper income groups or four different age categories. We will extract all relevant data and discuss as a team to decide how best to approach the analysis. This will be decided in the context of the available data from other studies to ensure we are including homogeneous behaviour/determinant combinations in meta‐analysis. For example, we would seek to avoid a situation where one study defines ‘young people’ vs ‘older people’ as under 30 s versus over 30 s with a sample aged 16–50 with another study that defines younger vs older as under 50 s versus over 50 s.

##### Dealing with missing data

If data is missing due to drop out from a study, we will use metrics where missing data were imputed, where reported. If not reported we will include the data but consider the effect of inclusion of studies with more than 20% attrition in sensitivity analysis. We will also include cross‐sectional studies where authors have weighted data to account for skew in the sample sociodemographic compared to the population of interest.

If study reports do not contain sufficient data to allow calculation of effect size estimates we will contact authors to obtain necessary data.

##### Assessment of heterogeneity

Heterogeneity will be assessed first, through visual inspection of the forest plot and checking for overlap of confidence intervals and second through the *Q*, *I* ^2^ and *τ* ^2^ statistics. Sources of heterogeneity that we anticipate are; differences in the populations studied, differences in the disease of interest, geographical location and phase of the outbreak at the time of data collection.

##### Assessment of reporting biases

A funnel plot and Egger's linear regression test will be included to check for publication bias across included studies (Sterne et al., 2019). Where the funnel plot is asymmetrical, this indicates either publication bias or bias that relates to smaller studies showing larger treatment effects. The trim and fill method will be used where the funnel plot is asymmetrical (Higgins et al., 2020). This is a nonparametric technique that removes the smaller studies causing irregularity until there is a new symmetrical pooled estimate. The eliminated studies are then filled back in to reflect the new estimate. We anticipate that we will have sufficient studies (more than 10) and are mindful of the need for effect sizes to be heterogeneous for this test to have sufficient power. The results of this statistical test will be used along with visual inspection of the funnel plot and interpreted with caution.

##### Data synthesis

Given the diverse range of behaviour/determinant relationships that will have been investigated, we intend to use random effects models, using inverse‐variance estimation, for pairwise meta‐analysis of ORs. The analysis will be conducted using R and the range of commands externally developed to conduct meta‐analysis with R such as metafor. We intend to conduct separate meta‐analyses for each behaviour of interest with an additional analysis for composite measures of general protective behaviours.

##### Subgroup analysis and investigation of heterogeneity

We anticipate that behaviour/determinant relationships are likely to vary for a variety of reasons and we intend to investigate the following possibilities through subgroup analysis.

- Method of measuring behaviour—Does the way in which behaviour is measured impact the strength of the relationship between determinants and behaviour? We will code effects into three categories depending on the method of measuring behaviour; observed behaviour, self‐report of current/recent behaviour and self‐report of imagined or intended future behaviour.
- PROGRESS‐PLUS characteristics—does the relationship between the behaviour and determinant vary depending on place of residence, race/ethnicity, occupation, gender or sex, religion, education, socioeconomic status or social capital. If subgroup analysis is not possible due to insufficient data we will endeavour to provide a brief narrative synthesis of any evidence on how these characteristics may interact with malleable psychosocial determinants to influence health protective behaviour.

We will conduct subgroup analyses for each of the factors above (method of measuring behaviour and PROGRESS‐PLUS characteristics) for each of the meta‐analyses. The subgroup analyses (based upon random‐effects models), will group studies by subcategory and estimate overall effects sizes for each. Based on the scoping search we anticipate that we will have a large number of studies and that there will be sufficient variability between studies to be confident that the subgroup analysis reflects differences in the strength of the relationship rather than differences between studies on some other confounding variable.

##### Sensitivity analysis

To test the effect of decisions made in the course of this review we will conduct the following sensitivity analysis for each of the index behaviours separately:

- Methodology: cross‐sectional versus designs where direction/causality can be inferred, for example, longitudinal or intervention studies with a control group.
- Studies that appear to exert undue influence on the findings. We will first check that there has not been a mistake in data extraction (e.g., SE mistaken for SD) before we analyse the effect of removing ‘outliers’.
- The quality of studies: we will assess the effect of removing the lowest quality studies from our analyses.

##### Treatment of qualitative research

Due to our limited resources we will not include qualitative studies in this review. However, we will include qualitative studies identified in our searches in the EGM.

### 3.6 Analysis and presentation

#### 3.6.1 Summary of findings table

We will summarise the results in a summary of findings table, with one row for each outcome (i.e., each health protective behaviour) determinant pairing, organised by outcome. The column headings will be: Strength of the evidence (with subheadings for number of studies, total number of participants, overall quality of included studies), summary of meta‐analysis if conducted or, if not, a brief narrative describing the findings in one or two sentences.

#### 3.6.2 EGM

The review team will import all relevant research to EPPI‐Reviewer software (Thomas et al., 2010) and all relevant data will be extracted to generate a live, accessible and interactive map using EPPI‐mapper software.

### 3.7 Stakeholder engagement

The review questions were developed through consultation with the Behaviour Change Group formed in response to COVID‐19 by the Public Health Agency, Northern Ireland. The group consists of public health officials and academic experts.

In addition, we will convene an advisory group consisting of international experts on evidence synthesis, behaviour change, public health and members of the public recruited through established fora for public involvement in science.

To develop an EGM framework that best represents research on determinants of COVID‐19 related behaviours, the specifics of the framework will be developed in consultation with key stakeholders involved in the advisory group. This framework will form the basis of the data extraction for visual presentation of the included evidence. However, we will follow the standard EGM framework as a matrix with rows representing the behaviours of interest and columns representing the determinants of behaviour. The map will also include information filters such as the study design and geographical context.

### 3.8 Conceptual framework

We deliberately do not approach this synthesis with a particular framework or theory of health behaviour driving the selection of potential determinants or synthesis decisions. Instead, we seek to produce a list of potential malleable determinants that could be changed by public health intervention. That way, we can provide a summary of the published evidence on which factors do and do not determine behaviours of interest. For those malleable factors that do relate to behaviours of interest, we will quantify the strength of the relationship through meta‐analysis.

## CONTRIBUTIONS OF AUTHORS

The review will be undertaken by a team with substantial expertise in systematic reviews, health behaviour and infectious diseases. Professor Martin Dempster will be the Principal Investigator (PI) of the project and will have overall responsibility for its conduct and delivery. Drs Ciara Keenan and Jennifer Hanratty will be responsible for the day‐to‐day operation of the review and will act as information retrieval specialists and lead screening, data extraction, quality assessment and reporting. Professor Miller will support the analysis and contribute to screening, data extraction, quality assessment and reporting. Dr Bradley is the content expert on communicable diseases and will also contribute to screening, data extraction, quality assessment and reporting.

Dr Jennifer Hanratty is a psychologist and expert in evidence synthesis. She has worked in evidence synthesis since 2012 and published reviews with Campbell, Cochrane and NIHR Health Technology Assessment amongst others. Jennifer is editor with Campbell Education Co‐ordinating group, Fellow with Campbell UK & Ireland and an invited member of the advisory board for Evidence Synthesis Ireland.

Dr Ciara Keenan is a methods editor and information retrieval specialist for the Campbell collaboration. She has considerable experience conducting and leading the creation of EGMs and systematic reviews. Ciara will run all searches with input from key stakeholder and co‐authors and will ensure that all methods adhere to the current Campbell MECCIR guidance for Systematic Reviews and Evidence and Gap Maps.

Professor Sarah Miller is Director of Campbell UK & Ireland. She is co‐chair and co‐editor of the Campbell Education Coordinating Group and Deputy Director of the Centre for Evidence and Social Innovation. She has considerable methodological and statistical expertise, which includes the conduct and analysis of randomised controlled trials as well as systematic reviews and meta‐analyses.

Dr Declan Bradley is a consultant in public health medicine and clinical lecturer in public health. He worked as a consultant in health protection (communicable disease control) before taking up his current post. His publishing record includes several systematic reviews and studies of healthcare‐related behaviour. He is a member of the Northern Ireland COVID‐19 Modelling and Behaviour Change Groups.

Dr Sean O'Connor is a Physiotherapist and an experienced health care researcher. He has undertaken a number of systematic reviews and studies related to behavioural interventions in the context of COVID‐19. He has an extensive knowledge of theory‐based implementation models for maximising integration of evidence into practice, systematic review methods including methodological quality/risk of bias assessment and the examination of stakeholder perspectives in healthcare delivery. Professor Martin Dempster, is a registered Health Psychologist, with over 20 years' experience in conducting research on the determinants of behaviour change. He has published 14 reviews, including reviews of effectiveness and reviews of covariates (as proposed in the current project). He has also published on the methodology of reviews. Dr Dempster is also a Chartered Statistician, with expertise in conducting meta‐analyses. Furthermore, he is currently a member of the Northern Ireland Public Health Agency COVID‐19 Behaviour Change Group, and is actively involved in leading health psychology groups within the British Psychological Society. He is, therefore, well placed to identify relevant evidence, contact those working on ongoing projects, and disseminate the findings of this project.

- Content: Bradley, Dempster, Hanratty, Miller, Keenan, O'Connor
- Systematic review methods: Hanratty, Miller, Dempster, Keenan, O'Connor.
- Statistical analysis: Dempster, Miller, Hanratty, Keenan, O'Connor
- Qualitative Evidence Synthesis: n/a.
- Information retrieval: Hanratty, Keenan.

## DECLARATIONS OF INTEREST

The authors declare no real or perceived conflict of interest with respect to the research, authorship, and/or publication of the reviews.

## PLANS FOR UPDATING THE EGM

Our approach is to conduct a series of reviews simultaneously and we aim to maintain the reviews as ‘living' systematic reviews for the 18 month project (till October 2022). We are seeking further resources to maintain the EGM and keep the reviews up to date in the context of COVID and/or future similar outbreaks.

## SOURCES OF SUPPORT

External sources

• New Source of support, UK

UKRI COVID rolling cal

## Supporting information

Supporting information.

## References

[cl21219-bib-0001] Cucinotta, D. , & Vanelli, M. (2020). WHO declares COVID‐19 a pandemic. Acta bio‐medica : Atenei Parmensis, 91(1), 157–160. 32191675 10.23750/abm.v91i1.9397 PMC7569573

[cl21219-bib-0002] Flumignan, R. L. G. , Nakano, L. C. U. , Pascoal, P. I. F. , Santos, B. C. D. , Correia, R. M. , Silveira, B. P. , Takihi, F. A. , Flumignan, C. D. Q. , Amorim, J. E. , & Atallah, Á. N. (2020). Evidence from Cochrane systematic reviews for controlling the dissemination of COVID‐19 infection. A narrative review. São Paulo Medical Journal = Revista paulista de medicina, 138(4), 336–344. 32638939 10.1590/1516-3180.2020.029105062020 PMC9673837

[cl21219-bib-0003] Gartlehner, G. , Affengruber, L. , Titscher, V. , Noel‐Storr, A. , Dooley, G. , Ballarini, N. , & König, F. (2020). Single‐reviewer abstract screening missed 13 percent of relevant studies: a crowd‐based, randomized controlled trial. Journal of Clinical Epidemiology, 121, 20–28. 31972274 10.1016/j.jclinepi.2020.01.005

[cl21219-bib-0004] Girum, T. , Lentiro, K. , Geremew, M. , Migora, B. , Shewamare, S. , & Shimbre, M. S. (2021). Optimal strategies for COVID‐19 prevention from global evidence achieved through social distancing, stay at home, travel restriction and lockdown: A systematic review. Archives of Public Health, 79(1), 150. 34419145 10.1186/s13690-021-00663-8 PMC8380106

[cl21219-bib-0005] Hedges, L. V. , Tipton, E. , & Johnson, M. C. (2010). Robust variance estimation in meta‐regression with dependent effect size estimates. Research Synthesis Methods, 1(1), 39–65. 26056092 10.1002/jrsm.5

[cl21219-bib-0006] Higgins, J. , Thomas, J. , Chandler, J. , Cumpston, M. , Li, T. , Page, M. , & Welch, V. (2020). Cochrane Handbook for Systematic Reviews of Interventions. Cochrane.

[cl21219-bib-0007] Houghton, C. , Meskell, P. , Delaney, H. , Smalle, M. , Glenton, C. , Booth, A. , Chan, X. H. S. , Devane, D. , & Biesty, L. M. (2020). Barriers and facilitators to healthcare workers' adherence with infection prevention and control (IPC) guidelines for respiratory infectious diseases: a rapid qualitative evidence synthesis. Cochrane Database of Systematic Reviews, 21(4), CD013582. 10.1002/14651858.CD013582 PMC7173761 32315451

[cl21219-bib-0008] Jefferson, T. , Del Mar, C. B. , Dooley, L. , Ferroni, E. , Al‐Ansary, L. A. , Bawazeer, G. A. , van Driel, M. L. , Jones, M. A. , Thorning, S. , Beller, E. M. , Clark, J. , Hoffmann, T. C. , Glasziou, P. P. , & Conly, J. M. (2020). Physical interventions to interrupt or reduce the spread of respiratory viruses. Cochrane Database of Systematic Reviews, 11(11), Cd006207. 10.1002/14651858.CD006207.pub5 PMC8094623 33215698

[cl21219-bib-0009] Levay, P. , & Finnegan, A. (2021). The NICE COVID‐19 search strategy for Ovid MEDLINE and Embase: developing and maintaining a strategy to support rapid guidelines. medRxiv 2021.06.11.21258749, 1, 1–61.

[cl21219-bib-0010] Michie, S. , & West, R. (2020). Behavioural, environmental, social, and systems interventions against covid‐19. BMJ, 370, m2982. 32723732 10.1136/bmj.m2982

[cl21219-bib-0011] Noel‐Storr, A. , Dooley, G. , Affengruber, L. , & Gartlehner, G. (2021). Citation screening using crowdsourcing and machine learning produced accurate results: Evaluation of Cochrane's modified Screen4Me service. Journal of Clinical Epidemiology, 130, 23–31. 33007457 10.1016/j.jclinepi.2020.09.024

[cl21219-bib-0012] Noel‐Storr, A. , Dooley, G. , Elliott, J. , Steele, E. , Shemilt, I. , Mavergames, C. , Wisniewski, S. , McDonald, S. , Murano, M. , Glanville, J. , Foxlee, R. , Beecher, D. , Ware, J. , & Thomas, J. (2021). An evaluation of Cochrane Crowd found that crowdsourcing produced accurate results in identifying randomized trials. Journal of Clinical Epidemiology, 133, 130–139. 33476769 10.1016/j.jclinepi.2021.01.006

[cl21219-bib-0013] Noel‐Storr, A. H. , Redmond, P. , Lamé, G. , Liberati, E. , Kelly, S. , Miller, L. , Dooley, G. , Paterson, A. , & Burt, J. (2021). Crowdsourcing citation‐screening in a mixed‐studies systematic review: A feasibility study. BMC Medical Research Methodology, 21(1), 88. 33906604 10.1186/s12874-021-01271-4 PMC8077753

[cl21219-bib-0014] Seto, W. H. , Tsang, D. , Yung, R. W. , Ching, T. Y. , Ng, T. K. , Ho, M. , Ho, L. M. , & Peiris, J. S. (2003). Effectiveness of precautions against droplets and contact in prevention of nosocomial transmission of severe acute respiratory syndrome (SARS). Lancet, 361(9368), 1519–1520. 12737864 10.1016/S0140-6736(03)13168-6 PMC7112437

[cl21219-bib-0015] Shea, B. J. , Reeves, B. C. , Wells, G. , Thuku, M. , Hamel, C. , Moran, J. , Moher, D. , Tugwell, P. , Welch, V. , Kristjansson, E. , & Henry, D. A. (2017). AMSTAR 2: A critical appraisal tool for systematic reviews that include randomised or non‐randomised studies of healthcare interventions, or both. BMJ, 358, j4008. 28935701 10.1136/bmj.j4008 PMC5833365

[cl21219-bib-0016] Sterne, J. A. , Hernán, M. A. , Reeves, B. C. , Savović, J. , Berkman, N. D. , Viswanathan, M. , Henry, D. , Altman, D. G. , Ansari, M. T. , Boutron, I. , Carpenter, J. R. , Chan, A. W. , Churchill, R. , Deeks, J. J. , Hróbjartsson, A. , Kirkham, J. , Jüni, P. , Loke, Y. K. , Pigott, T. D. , … Higgins, J. P. (2016). ROBINS‐I: A tool for assessing risk of bias in non‐randomised studies of interventions. BMJ, 355, i4919. 27733354 10.1136/bmj.i4919 PMC5062054

[cl21219-bib-0017] Sterne, J. A. C. , Savović, J. , Page, M. J. , Elbers, R. G. , Blencowe, N. S. , Boutron, I. , C. J. , Cheng, H. Y. , Corbett, M. S. , Eldridge, S. M. , Emberson, J. R. , Hernán, M. A. , Hopewell, S. , Hróbjartsson, A. , Junqueira, D. R. , Jüni, P. , Kirkham, J. J. , Lasserson, T. , Li, T. , … Cates (2019). RoB 2: A revised tool for assessing risk of bias in randomised trials. BMJ, 366(Suppl 1), 1–56. 10.1136/bmj.l4898 31462531

[cl21219-bib-0018] The Joanna Briggs Institute . (2017). *The Joanna Briggs Institute Critical Appraisal tools for use in JBI Systematic Reviews. Checklist for Analytical Cross Sectional Studies*.

[cl21219-bib-0019] The Joanna Briggs Institute . (2020). *The Joanna Briggs Institute Critical Appraisal tools for use in JBI Systematic Reviews. Checklist for Cohort Studies*.

[cl21219-bib-0020] Thomas, J. , Brunton, J. , & Graziosi, S. (2010). EPPI‐Reviewer 4.0: Software for research synthesis. EPPI‐Reviewer 4.0. London: EPPI‐Centre Software, Social Science Research Unit, Institute of Education, University of London.

[cl21219-bib-0021] Tipton, E. (2013). Robust variance estimation in meta‐regression with binary dependent effects. Research Synthesis Methods, 4(2), 169–187. 26053656 10.1002/jrsm.1070

[cl21219-bib-0022] Tipton, E. , & Pustejovsky, J. E. (2015). Small‐sample adjustments for tests of moderators and model fit using robust variance estimation in meta‐regression. Journal of Educational and Behavioral Statistics, 40(6), 604–634.

[cl21219-bib-0023] Warren‐Gash, C. , Fragaszy, E. , & Hayward, A. C. (2013). Hand hygiene to reduce community transmission of influenza and acute respiratory tract infection: A systematic review. Influenza and Other Respiratory Viruses, 7(5), 738–749. 23043518 10.1111/irv.12015 PMC5781206

[cl21219-bib-0024] Webster, R. K. , Brooks, S. K. , Smith, L. E. , Woodland, L. , Wessely, S. , & Rubin, G. J. (2020). How to improve adherence with quarantine: Rapid review of the evidence. Public Health, 182, 163–169. 32334182 10.1016/j.puhe.2020.03.007 PMC7194967

[cl21219-bib-0025] West, R. , Michie, S. , Rubin, G. J. , & Amlôt, R. (2020). Applying principles of behaviour change to reduce SARS‐CoV‐2 transmission. Nature Human Behaviour, 4(5), 451–459. 10.1038/s41562-020-0887-9 32377018

[cl21219-bib-0026] White, H. , Bianca, A. , Gaarder, M. , Kornør, H. , Littell, J. , Marshall, Z. , Matthew, C. , Pigott, T. , Snilstveit, B. , Waddington, H. , & Welch, V. (2020). Guidance for producing a Campbell evidence and gap map. Campbell Systematic Reviews, 16(4), e1125. 10.1002/cl2.1125 PMC8356343 37016607

[cl21219-bib-0027] Wu, F. , Zhao, S. , Yu, B. , Chen, Y. M. , Wang, W. , Song, Z. G. , Hu, Y. , Tao, Z. W. , Tian, J. H. , Pei, Y. Y. , Yuan, M. L. , Zhang, Y. L. , Dai, F. H. , Liu, Y. , Wang, Q. M. , Zheng, J. J. , Xu, L. , Holmes, E. C. , & Zhang, Y. Z. (2020). A new coronavirus associated with human respiratory disease in China. Nature, 579(7798), 265–269. 32015508 10.1038/s41586-020-2008-3 PMC7094943

