# Supplementary material for: PROTOCOL: Psychological and psychosocial determinants of COVID Health Related Behaviours (COHeRe): A suite of systematic reviews and an evidence and gap map
Source: Campbell Syst Rev. 2022 Feb 3;18(1):e1219. doi: 10.1002/cl2.1219 (PMC8812813; doi:10.1002/cl2.1219)
Supplement: Supplementary file 1 — Supporting information. [file CL2-18-e1219-s001.docx]

# Appendices

## 1 Data extraction Sheet for COHeRe Review

| Study ID | First author surname year |
| --- | --- |
| Title | Copy & paste title |
| Country | Name of country |
| Study design | Select from dropdown list  Cross-sectional  Longitudinal  Intervention |
| Timing | List- When was data was collected? Before, during, after or hypothetical  Hypothetical  Pre-pandemic  During pandemic  After-pandemic  Before and after  Before and during  During and after  Before, during and after |
| When? | When was the study conducted - date given and any other relevant info on the time frame |
| Sample size | Number of participants included |
| Other info | Note page number of direct quotes |
| Reviewer | Who extracted the data? Enter initials |
| Checked by | Who checked the accuracy of the data extraction? Enter initials |
| Excluded? | State reason for exclusion |

|  | **Population** |
| --- | --- |
| Study ID | Author year |
| Where recruited from? | List  Administrative data  General public  Hospital/Clinical Setting  Patient group  In isolation/quarantine  Mass gathering/event  Other  Schools  University/colleges  Not reported |
| Describe the population | Describe the population studied and any inclusion and exclusion criteria for the study, so that the reader can understand who is being studied  (e.g. older adults, psychology students, prmary school children) |
| Sectors | Were the participants asked about preventative behaviours in any of the following sectors?. List  Work  Retail  Education  Travel  Family & community  Sport, culture, leisure  No specific sectors were identified  Other – specify |
| Geographic location | List  1. Urban  2. Rural  3. Suburban  4. Mixed  5. Other  Not reported |
| Age group | Text |
| Age detail | Mean age (SD) range |
| Gender | % female |
|  | n female |
|  | % male |
|  | n male |
| Ethnicity | Text |
| Socio-economic status | Extract any information on socioeconomic status |
| Other |  |
| Disease status | List  1. Tested positive  2. Suspected  3. Tested negative  4. Not suspected  Not Specified |
| Disease status details | if other give details |

| Study ID | | First author + year | |
| --- | --- | --- | --- |
| **Behaviour** | | | |
| Preventative Behaviour | | List  **Behaviour**  **Description of behaviours**  Handwashing  Washing hands more frequently with soap and water or the use of hand sanitizer     if handwashing facilities are not available  Masks /face covering  Wearing any type of mask or face covering. This can include medical grade masks, face shields, homemade masks, covering face with a scarf etc.  Physical Distancing  Maintaining the recommended distance from others when physically present. The recommended distance varies by setting but is typically in the region of 1 to 3 meters.  Social Distancing  Minimising social contact with those outside of your own household. This is a very broad category and includes working from home, avoiding crowded places, only leaving home when necessary (e.g. to purchase food or medicines) and not socialising with others in your own home or garden.  Isolation /quarantine  Self-isolation and/or quarantine refers to keeping separate from all other people either because you have or are suspected to have the virus. Self-isolation is typically voluntary but often recommended by the government/health authorities. Quarantine is typically enforced in either a mandated setting, one's own home, or temporary accommodation for those in travelling away from home.  Respitory hygiene/ ettiquette  Includes tissue hygiene, which means using a tissue to cover nose and mouth when coughing, sneezing or blowing your nose and immediately disposing of the tissue. When tissues are not available coughing/sneezing into your elbow and not your hands.  Cleaning surfaces  Disinfecting high touch surfaces in home and office/retail/public spaces or items brought into the home.  Avoiding t-zone  Avoiding touching your face specifically the t-zone; eyes, nose & mouth  Other  Other analogous relevant behaviours or aggregate measures of multiple relevant behaviours | |
| Description | | Brief description of behaviour as reported in the study | |
| When? | | Reporting time frame  Retrospective reporting  Current behaviour  diary methods, reporting on today or observation of behaviour in real time.  Prospective/hypothetical  report on intended or anticipated future behaviour | |
| Who? | | Who collected the information?  1. Self-report  2. Family-report  3. Clinician/medical profession  4. Independent observer  5. Other  Not Specified | |
| Type? | | Type of Measurement  1. Clinical (direct assessment perhaps through administrative recored)  2. Self/other Report (questionnaire or survey)  3. Physical Specimens  4. Observation  5. Structured Interview  6. Other (please specify)  7. Not Specified | |
| Quality? | | Were the measurement tools reported as reliable/valid?  Yes - reliable  No - not reliable  Not reported  Not applicable | |
| Direction? | | Direction of scales  Higher score is good/more is better  Lower score is good/less is better  NA | |
| **Determinant** | | | |
| Description | | Brief description of determinant as reported in the study | |
| When? | | Reporting time frame (list as above) | |
| Who? | | Who collected the information? (list as above) | |
| Type? | | Type of Measurement (list as above) | |
| Quality? | | Were the measurement tools reported as reliable/valid? (list as above) | |
| Direction? | | Direction of scales (list as above) | |
| Adjusted or unadjusted? | | Speify weather the data is adjusted or unadjusted and what variables were accounted for in the analysis | |
| **Behaviour/Determinant relationship quantitative data** | | | |
| Summary | Description of findings | | Narrative description of findings (copy paste from paper or summarise in own words) |
| n | n | | sample size for this specific analysis |
| Data - complete relevant fields only | r | | correl |
|  | d | | cohens d |
|  | OR | | odds ratio or adjusted odds ratio |
|  | B | | regression coefficient (adjusted or not) |
|  | SDIV | | Standard deviation of IV |
|  | SDDV | | Standard deviation of DV |
|  | p | | P value reported |
|  | Lower CI | | Lower confidence interval |
|  | Upper CI | | Upper confidence interval |
|  | N w | | N with determinant |
|  | N wo | | N without determinant |
|  | E w | | Events with determinant |
|  | E wo | | Events without determinant |
|  | F | | F |
|  | df2F | | df2F |
|  | t | | t |
|  | dft | | dft |
|  | Mean1 | | Mean1 |
|  | Mean2 | | Mean2 |
|  | SE1 | | SE1 |
|  | SE2 | | SE2 |
|  | SD1 | | SD1 |
|  | SD2 | | SD2 |
|  | n1 | | n1 |
|  | n2 | | n2 |
|  |  |  |  |

## 2 Modifications to the JBI tool for assessing risk of bias/quality in cross-sectional studies

| **Original tool** | **Modified/additional items** |
| --- | --- |
| 1. Were the criteria for inclusion in the sample clearly defined? | 1a. Were the criteria for inclusion in the sample clearly defined **and adhered to**? |
|  | 1b. Was the sample randomly selected from the defined population? |
| 2. Were the study subjects and the setting described in detail? | 2. Was the sample included in the study representative of the population of interest? |
| 3. Was the exposure measured in a valid and reliable way? | 3. Were the **determinants** measured in a valid and reliable way? |
| 4. Were objective, standard criteria used for measurement of the condition? | 4a.. Were objective, standard criteria used for measurement of the **behaviours** of interest? |
|  | 4b. Were the **behaviours** measured in a valid and reliable way? |
| 5. Were confounding factors identified? | 5. Were confounding factors /**coviariates** identified? |
| 6. Were strategies to deal with confounding factors stated? | 6. Were strategies to deal with confounding factors/ **covariates** stated **and used**? |
| 7. Were the outcomes measured in a valid and reliable way? | no change |
| 8. Was appropriate statistical analysis used? | no change |
